# Situating pain in a more helpful place

**DOI:** 10.1097/PR9.0000000000000642

**Published:** 2018-03-05

**Authors:** Mike Osborn

**Affiliations:** Royal United Hospitals NHS Foundation Trust, Bath, United Kingdom

## Abstract

Milton Cohen et al. proposed a revision of the IASP definition of pain of 1979. This commentary summarizes, why this redefinition is necessary, appropriate, and timely.

**Commentary on**: Cohen M, Quintner J, van Rysewyk S. Reconsidering the IASP definition of pain. PAIN Reports 2018:e634.

**See also**: Treede R-D. The IASP definition of pain: as valid in 2018 as in 1979, but in need of regularly updated footnotes. PAIN Reports 2018:e643.

I approached this article by Cohen et al.^1^ with a real sense of anticipation as I have struggled with the current definition at times and the prospect of exploring a revised version was irresistible. I have never found the current definition very helpful in my clinical work and neither have many of the pain sufferers I have met. They often feel it does not capture their experience and can leave them more confused. I found this article very readable. My enthusiasm for it is not based on an absolute endorsement for the proposed new definition, but on the clarity of the writing, the willingness to engage in the slippery phenomenology of pain, and the potential to provoke debate. The article summarises the history of the definition of pain, outlines a range of compelling criticisms of the current definition and proposes a radical new one. The proposed definition struggles to discriminate between pain and other somatic experiences and to define the notion of a “mutual” experience but is probably already closer to the implicit, working definitions applied by practitioners and situates the pain experience in a more helpful place.

I was drawn most to the “*crisis of choice, action and of identity*” that the authors refer to early in the article. The crisis is for both clinician and patient and is a critical clinical issue. The associated risk of confusion and stigma^2^ remains a source of profound worry and disables the development of both understanding and accepting chronic pain. Most folk beliefs about chronic pain tend to fuel only disabling punitive explanations. A development of the definition could challenge these beliefs and better support the hard-pressed clinician who has to make eye contact with their patient but often cannot relieve their pain nor explain why in a culturally acceptable therapeutic way.^3^ The article includes one particular criticism of the current IASP definition related to the notes which still state that pain in the absence of a pathophysiological cause is possible and “*usually for psychological reasons*.” The authors argue, rightly I feel, that these notes show that the “*untenable concept of “psychogenic pain” is still extant*,” that folk beliefs inform the definition of pain to an unacceptable degree and this situates chronic pain in a threatening social context. The current definition does nothing to help resolve the “*crisis*” described by the authors and may even contribute to it. We need to improve the effectiveness of our pain services and in particular to know which elements have the most therapeutic potential and what is causing the most resistance to sense-making and adaptation.^4^ A revised definition of pain could help us ask the right questions and this article makes a positive contribution toward such a revision.

## Disclosures

The author has no conflict of interest to declare.
